# Beyond the Neutral Spine: A Narrative Review and Modern Framework for Low Back Injury Prevention in Deadlifting

**DOI:** 10.3390/sports14040151

**Published:** 2026-04-13

**Authors:** Bilel Cherni, Hamza Marzouki, Okba Selmi, Wesam Al Attar, Karim Chamari, Katsuhiko Suzuki

**Affiliations:** 1 Research Unit: Sport Sciences, Health and Movement, University of Jendouba, Kef 7100, Tunisia; bilelcherny@gmail.com (B.C.); hamzic_30@hotmail.com (H.M.); 2 High Institute of Sport and Physical Education of Kef, University of Jendouba, Kef 7100, Tunisia; 3 Department of Medical Rehabilitation Sciences, College of Applied Medical Sciences, Umm Al-Qura University, Makkah 24381, Saudi Arabia; wsattar@uqu.edu.sa; 4 High Institute of Sport and Physical Education of Ksar-Said, University of Manouba, Tunis 2010, Tunisia; karim.chamari@naufar.com; 5 Naufar Center, Doha P.O. Box 93097, Qatar; 6 Faculty of Sport Sciences, Waseda University, Tokorozawa 359-1192, Japan

**Keywords:** core stability, movement variability, strength training, injury prevention, neuromuscular control, spinal biomechanics

## Abstract

Traditional deadlift guidelines prioritize maintaining a neutral spine to prevent low back injuries. However, recent evidence questions whether moderate spinal flexion under load is inherently harmful, especially among trained individuals. This article proposes a modern, multifactorial framework for deadlift-related injury prevention that moves beyond rigid postural prescriptions. It integrates biomechanical evidence, load management strategies, movement variability principles, and dynamic trunk control. This narrative review synthesizes literature identified through structured searches of PubMed, Scopus, and Google Scholar, prioritizing peer-reviewed studies examining spinal biomechanics, load management, motor control, and injury epidemiology. Evidence suggests that trained lifters often exhibit natural lumbar flexion without clear prospective evidence of increased injury risk. Abrupt increases in training load appear to be consistently associated with elevated injury incidence, although relationships remain probabilistic and context-dependent. While technical factors, including spinal posture, may influence local tissue loading, current evidence suggests that rapid changes in training exposure and cumulative load management appear to be more consistent predictors of injury risk than isolated deviations from an externally defined “neutral” alignment. Movement variability appears protective, and dynamic trunk control is more functionally relevant than static core strength. A paradigm shift is needed in how deadlifts are coached and programmed. Injury prevention should emphasize progressive loading, adaptive movement strategies, and dynamic stability, rather than rigid technique enforcement. Rather than systematically appraising all available evidence, this review offers an interpretative synthesis to guide modern, evidence-informed coaching and rehabilitation practice.

## 1. Introduction

Deadlifting is widely recognized as a foundational exercise in strength training and athletic conditioning, owing to its effectiveness in developing posterior chain musculature, enhancing neuromuscular coordination, and transferring to functional and sport-specific tasks [1]. As a multi-joint, closed-chain movement involving substantial external loads, the deadlift has long been central to both performance-oriented training and rehabilitation contexts [1,2]. However, despite its benefits, the deadlift is frequently associated with concerns regarding low back injury, particularly among recreational lifters and strength athletes [2]. These concerns have historically shaped coaching practices and injury prevention strategies, most notably through the widespread promotion of maintaining a strictly neutral spine throughout the movement [3]. The neutral spine paradigm is rooted in biomechanical models suggesting that spinal flexion under load increases intervertebral disk strain and shear forces, thereby elevating injury risk [3]. Consequently, technical instruction in resistance training has traditionally emphasized minimizing lumbar motion and maintaining a rigid spinal posture during lifting tasks. While this approach has intuitive appeal and may be appropriate in certain populations (e.g., novices or individuals with acute pain), emerging evidence indicates that such a binary interpretation of spinal safety may oversimplify the complex and adaptive nature of human movement [4,5]. Recent biomechanical investigations demonstrate that even experienced lifters exhibit measurable degrees of lumbar flexion during heavy deadlifts, particularly at higher loads or during the transition between concentric and eccentric phases [6]. These findings challenge the long-standing assumption that deviations from neutral alignment are inherently pathological.

Indeed, accumulating evidence suggests that controlled spinal flexion under load does not necessarily correspond to increased injury risk when training exposure is appropriately managed [6,7]. For example, Christophy et al. [8] described dynamic spinal adjustments throughout lifting movements, indicating that spinal posture is not static but continuously regulated in response to external load and task demands. Similarly, systematic reviews examining the relationship between lumbar flexion and low back pain during lifting have failed to establish a direct causal link between moderate flexion and injury [9]. It is important to acknowledge that experimental and modeling studies have demonstrated increased disk strain and annular stress under repeated or extreme flexion loading, particularly under high compressive forces [8,10,11]. However, much of this evidence derives from cadaveric models, short-duration laboratory tasks, or biomechanical simulations rather than long-term prospective injury studies. Consequently, extrapolation to real-world training contexts requires caution [8]. Such findings support a reconceptualization of spinal mechanics during resistance training as a dynamic and context-dependent phenomenon, in which spinal posture represents one of several interacting contributors to tissue stress rather than a rigid or universally dominant determinant of injury [9].

Beyond spinal posture itself, contemporary models of musculoskeletal injury increasingly emphasize the dominant role of training load exposure. Sudden increases in volume or intensity, rather than isolated biomechanical variables, have consistently been identified as major predictors of injury risk across sporting populations [12,13]. In resistance training contexts, repetitive exposure to high compressive and shear forces may exceed tissue tolerance when progression is poorly managed, regardless of whether technique appears externally “correct” [6]. This perspective aligns with broader workload–injury frameworks, which view injury as the result of a mismatch between applied mechanical stress and an individual’s adaptive capacity [14,15]. Accordingly, injury prevention strategies that focus exclusively on posture while neglecting load management may fail to address the primary driver of tissue failure.

In parallel, growing attention has been directed toward the role of movement variability in injury resilience. Traditional coaching approaches often aim to minimize variability in movement execution, treating deviations from a prescribed technique as errors to be corrected. However, motor control theories increasingly conceptualize variability as an adaptive feature that allows biological systems to distribute mechanical stress across tissues and to respond flexibly to changing task demands [16,17]. Empirical evidence suggests that controlled variability in joint kinematics and coordination patterns may reduce repetitive strain and enhance long-term tissue tolerance [18]. In the context of deadlifting, subtle variations in trunk angle, hip strategy, or bar path may therefore reflect functional adaptability rather than technical deficiency.

Similarly, prevailing approaches to core training have been largely influenced by static stabilization concepts, emphasizing exercises such as planks or abdominal bracing drills as prerequisites for safe lifting [19,20]. In the context of this review, movement variability refers to trial-to-trial fluctuations in joint kinematics, coordination patterns, or force distribution during repeated task execution [16,17]. Dynamic trunk control refers to the neuromuscular capacity to regulate spinal alignment and trunk stiffness during externally loaded, multi-planar movement [21]. These constructs are typically quantified in the literature through kinematic variability metrics, coordination phase analysis, EMG activity patterns, and perturbation-response paradigms [22]. While these exercises can increase trunk muscle activation, their transfer to dynamic, multi-planar lifting tasks remains limited [23]. More recent frameworks define core stability not as static rigidity but as the neuromuscular capacity to maintain functional spinal control under perturbation and load [21]. This distinction has important implications for injury prevention, as dynamic trunk control more closely reflects the demands imposed by heavy deadlifts and other resistance exercises [24].

Psychological and perceptual factors further complicate the relationship between lifting technique and injury. Fear-avoidance beliefs surrounding spinal flexion have been shown to alter movement strategies and increase muscular co-contraction, potentially elevating spinal loading and impairing motor efficiency [25,26]. Coaching narratives that portray the spine as inherently fragile may inadvertently reinforce maladaptive motor patterns and contribute to kinesiophobia. Conversely, educational approaches emphasizing spinal robustness and adaptability have been associated with improved lifting performance and more positive beliefs about back health [27]. These findings underscore the need to integrate cognitive and behavioral dimensions into biomechanical and physiological models of injury risk.

Despite these advances, many coaching and rehabilitation paradigms remain anchored in rigid postural prescriptions that prioritize maintaining spinal neutrality above all other considerations [28]. Such approaches may underestimate the importance of load progression, movement adaptability, and neuromuscular control, while overemphasizing externally observable alignment. This mismatch between traditional doctrine and contemporary evidence highlights the need for a more integrative framework for understanding low back injury risk in deadlifting.

Therefore, the purpose of this narrative review is to synthesize current biomechanical, neuromuscular, and training-related evidence relevant to low back injury prevention in deadlifting and to propose a modern, multifactorial conceptual framework that moves beyond rigid postural prescriptions. Specifically, this review integrates findings related to (i) spinal loading and flexion tolerance, (ii) training load management, (iii) movement variability, (iv) dynamic trunk control, and (v) psychological and coaching factors. Rather than providing a systematic or quantitative appraisal of all available studies, this article adopts an interpretive narrative approach aimed at contextualizing key findings within a coherent theoretical model.

Based on contemporary research on spinal biomechanics [29], thoracolumbar alignment [30], and workload–injury relationships [31], this review hypothesizes that emphasizing load tolerance, adaptability, and dynamic control, rather than strict adherence to a neutral spinal posture, may better support both spinal health and resistance training performance. By reframing deadlift-related injury prevention within an explicitly biopsychosocial and load-based paradigm, in which cognitive beliefs influence motor behavior, which in turn modifies mechanical loading, this work seeks to integrate psychological, neuromuscular, and biomechanical processes within a unified adaptive model.

## 2. Materials and Methods

This article was conducted as a narrative, interpretive review aimed at synthesizing and contextualizing current evidence related to low back injury prevention in deadlifting. A narrative approach was selected to allow for theoretical integration across biomechanical, neuromuscular, training, and psychological domains, rather than to provide an exhaustive or quantitative synthesis of the literature. This design was considered appropriate given the conceptual nature of the topic and the heterogeneity of study designs addressing spinal loading, motor control, and injury risk in resistance training.

### 2.1. Literature Search Strategy

A structured but non-exhaustive literature search was performed using the electronic databases PubMed, Scopus, and Google Scholar. The search included studies published from database inception to December 2025, with particular emphasis on literature from the past 15 years, while also incorporating seminal biomechanical studies that established foundational concepts in spinal loading and informed contemporary perspectives in biomechanics and strength training research. Searches were conducted using combinations of the following key terms: “deadlift biomechanics,” “lumbar spine,” “spinal flexion,” “neutral spine,” “core stability,” “movement variability,” “dynamic trunk control,” “load management,” and “injury prevention in strength training.” Boolean operators (AND/OR) were applied to refine results, and reference lists of relevant articles were manually screened to identify additional pertinent studies.

Priority was given to peer-reviewed articles published in English that examined (i) biomechanical loading of the spine during deadlifting or lifting tasks, (ii) relationships between spinal posture and low back pain or injury, (iii) training load and workload–injury associations, (iv) motor control and movement variability, and (v) neuromuscular and psychological factors relevant to trunk control and injury prevention. Both seminal publications and recent contributions (particularly from the last decade) were considered in order to integrate foundational theories with contemporary findings.

### 2.2. Study Selection and Thematic Organization

This selective approach allowed the integration of experimental, observational, and theoretical studies addressing different components of spinal health.

The retrieved literature was organized into five interrelated thematic domains:

1. natural spinal flexion and spinal loading during deadlifting,
2. training load management and workload-related injury risk,
3. movement variability and coordination strategies,
4. dynamic trunk control and core training paradigms, and
5. psychological and coaching-related influences on movement behavior and injury risk.

This thematic structure was used to guide the narrative synthesis and to support the development of an integrative conceptual framework for injury prevention.

### 2.3. Data Synthesis and Interpretive Framework

Given the narrative nature of this review, findings were synthesized qualitatively through interpretive analysis rather than through formal meta-analysis or risk-of-bias assessment. Evidence from each thematic domain was examined for converging and diverging patterns and was subsequently integrated into a unified conceptual model emphasizing the interaction between biomechanical loading, neuromuscular adaptability, training exposure, and cognitive–behavioral factors.

The synthesis focused on identifying mechanistic principles (e.g., load tolerance, adaptive variability, and dynamic stability) rather than on quantifying effect sizes. This approach enabled the interpretation of results within a broader biopsychosocial framework of injury risk and resilience, consistent with contemporary perspectives on musculoskeletal health.

### 2.4. Methodological Considerations

As a narrative review, this work does not aim to provide an exhaustive or systematic appraisal of all available evidence and may be subject to selection and interpretive bias. However, the goal of the present approach is not to establish causal estimates but to propose a coherent, theory-driven framework that integrates diverse strands of research relevant to deadlifting mechanics and low back injury prevention.

Given the narrative design of this review, the following sections represent a thematic synthesis of reported findings rather than a formal results aggregation with quantitative weighting. Evidence from biomechanical, epidemiological, and motor control studies is therefore interpreted within a conceptual framework rather than through statistical pooling.

#### 2.4.1. Natural Spinal Flexion in Deadlifting

While a rigid neutral spine remains a common coaching cue, findings from Howe and Lehman [31] and the systematic review by Saraceni et al. [9] suggest that lumbar spine flexion during lifting does not inherently increase the risk of pain or injury, underscoring the adaptability of the spine. Clinical guidelines by Delitto et al. [32] support the notion that spinal movement, including flexion, can be safely incorporated into rehabilitation and lifting strategies when guided by appropriate loading principles and functional goals. Ramirez et al. [6] reported modeled peak spinal compressive forces approaching 18 kN in men and approximately 8 kN in women during repetitive deadlifts under specific laboratory conditions. These values represent peak estimates derived from biomechanical modeling rather than direct in vivo measurements. For comparison, experimental and cadaveric studies have reported lumbar compressive tolerance ranges between approximately 6–10 kN for vertebral body failure and higher values for disk structures, although these thresholds vary substantially depending on age, bone density, loading rate, and specimen condition. Therefore, absolute threshold comparisons should be interpreted cautiously, as tissue tolerance is not a fixed universal value. Scott et al. [9] highlighted that spine education focused on resiliency improves deadlift performance, supporting controlled spinal flexion. Brandl et al. [33] also demonstrated that thoracolumbar fascia deformation during deadlifting differs between individuals with and without back pain, reinforcing the importance of spine adaptability rather than rigid posture.

To synthesize the available biomechanical evidence, Table 1 summarizes recent experimental studies investigating lumbar spine loading during deadlifting. These studies used motion capture, electromyography, and musculoskeletal modeling to quantify spinal compression, shear forces, trunk kinematics, and muscle activation patterns. Together, these investigations provide insight into the mechanical demands imposed on the lumbar spine during deadlift execution and highlight the potential mechanisms contributing to low-back injury risk.

Building on these findings, Table 2 presents experimental studies examining the influence of lumbar posture and spinal motion on mechanical loading during deadlift-type lifting tasks. These studies specifically investigated how trunk flexion, lumbar alignment, or fatigue-related posture changes affect spinal compression forces, shear loads, and lumbopelvic coordination. Understanding these relationships is central to the debate regarding the necessity of maintaining a strictly neutral spine during resistance training.

In addition, Table 3 summarizes studies comparing different deadlift variations, including conventional, sumo, and hex-bar deadlifts. These studies investigated how exercise selection and lifting technique influence trunk posture, joint moments, and spinal loading. Such comparisons are particularly relevant for injury prevention strategies, as different deadlift techniques may redistribute mechanical stress across the hip and lumbar spine.

#### 2.4.2. Load Management as a Primary Risk Factor

Strength training injuries, particularly in the lumbar region, correlate more strongly with workload spikes than with minor technical imperfections. Early workload research suggested that maintaining an acute: chronic workload ratio (ACWR) within a moderate range (e.g., ~0.8–1.3) may be associated with lower injury risk, whereas abrupt spikes in acute load relative to chronic exposure were linked to increased injury incidence [15]. Blanch and Gabbett [41] emphasized that rapid increases in training load are associated with higher injury rates, regardless of technical proficiency. Soligard et al. [13] endorsed these findings across sports, emphasizing the need for gradual progression, structured deload phases, and ongoing workload monitoring to foster tissue adaptation and resilience. However, the ACWR model has been subject to methodological debate. Critics have highlighted potential statistical artifacts related to ratio-based calculations, mathematical coupling, and inconsistent predictive performance across sports and populations [14,15]. Alternative approaches, including rolling averages, exponentially weighted moving averages, and absolute load metrics, have been proposed to address these limitations [14]. Accordingly, ACWR should not be interpreted as a definitive predictive tool, but rather as one operational attempt to quantify fluctuations in load exposure [14,15]. Schäfer et al. [29] found that managing mechanical spine loading during training is crucial to injury prevention.

Importantly, the central principle underlying workload–injury models is not the superiority of any single metric, but the concept that tissues adapt progressively to mechanical stress and may become vulnerable when load changes exceed adaptive capacity [13,15]. Within this framework, load management represents a dynamic interaction between exposure history, recovery, and individual tolerance rather than adherence to a specific numerical threshold [13,14].

#### 2.4.3. Movement Variability and Injury Resilience

It is important to acknowledge that most empirical work on movement variability derives from gait analysis, motor control theory, and overuse injury models rather than high-load resistance training specifically [16,17]. Direct longitudinal evidence linking variability in deadlifting technique to reduced lumbar injury incidence remains limited [18]. Therefore, the following discussion should be interpreted as a theoretically informed extrapolation rather than as direct proof of injury prevention effects in resistance training. Controlled variability in movement patterns has been proposed as a protective mechanism in overuse contexts, potentially by dispersing mechanical stress across tissues and reducing repetitive loading of identical structures [16,17,42]. Stergiou and Decker [17] argued that strategic, execution, and outcome variability enhance adaptability and reduce repetitive strain. By extrapolation, structured variations in trunk and hip mechanics during lifting may contribute to resilience, although this hypothesis has yet to be directly tested in longitudinal resistance training studies, as Seay et al. [18] suggest that movement coordination variability is associated with lower injury risk and greater adaptability. As highlighted by Bartlett et al. [16], elite performers naturally employ movement variability to adapt to task and environmental demands, suggesting that deliberate incorporation of variability in deadlift programming may bolster long-term tissue health. Thoracolumbar and lumbopelvic spinal alignment during deadlifting varies between men and women, emphasizing the need for individualized approaches in managing spinal alignment and injury prevention [30,43]. Future prospective studies examining whether variability-based programming influences lumbar injury rates in strength-trained populations are needed to substantiate this theoretical link.

#### 2.4.4. Dynamic Core Control vs. Static Core Training

In this review, “static core training” refers to isometric trunk stabilization exercises performed under minimal external perturbation (e.g., planks, side planks, abdominal bracing drills) [19,20]. “Dynamic trunk training” refers to exercises involving trunk movement, load transfer, or perturbation under external resistance (e.g., anti-rotation drills, loaded carries, cable-based trunk resistance exercises) [21,23]. These definitions are provided for conceptual clarity rather than as rigid categorical distinctions. Static core exercises have limited transfer to dynamic lifts [19]. Behm et al. [23] ad Haugen et al. [44] showed that anti-rotation drills and loaded carries produce greater trunk muscle activation under movement than static holds. Granacher et al. [24] and Hibbs et al. [20] demonstrated that a dynamic core stability program improved both spinal stability and resistance training performance in young athletes. Huxel Bliven and Anderson [21] emphasized that true core stability is the neuromuscular ability to maintain spinal equilibrium under perturbation, exactly the demand posed by heavy deadlifts.

The abdominal wall musculature, including the transversus abdominis, internal and external obliques, and rectus abdominis, contributes to intra-abdominal pressure generation and trunk stiffness regulation [19,20]. Both isolated strengthening and integrated compound exercises may enhance these capacities [21,23]. However, the extent to which isolated abdominal strengthening independently reduces lumbar injury risk during heavy deadlifting remains insufficiently established [21].

These findings suggest that dynamic, task-specific core training may complement static bracing capacity, particularly when preparing individuals for the coordinative and perturbation demands of heavy lifting [19,21]. Rather than representing competing paradigms, static and dynamic approaches may be integrated within a periodized framework depending on training stage, load magnitude, and individual needs [19,20]. Importantly, static bracing capacity likely plays a foundational role in force transmission, intra-abdominal pressure regulation, and early motor learning [17,18]. Dynamic trunk exercises should therefore be viewed not as replacements but as context-specific extensions that enhance adaptability under load [19,21].

#### 2.4.5. Psychological and Coaching Factors

Fear-avoidance beliefs surrounding any spinal flexion can paradoxically increase injury risk by promoting hypervigilance and excessive bracing [22,45]. O’Sullivan et al. [25] reported that powerlifters who internalize “no flexion” dogma often adopt maladaptive motor patterns. Liew et al. [26] found widespread kinesiophobia among strength athletes concerning everyday bending. Modern coaching should replace rigid posture policing with confidence-building cues (e.g., “drive through the heels”, “breathe”, and “move smoothly”), emphasizing intent and load tolerance over perfect technique. Scott et al. [27] further showed that resiliency-focused spine education improved both deadlift performance and back-related beliefs.

## 3. Discussion

**Figure 1:**
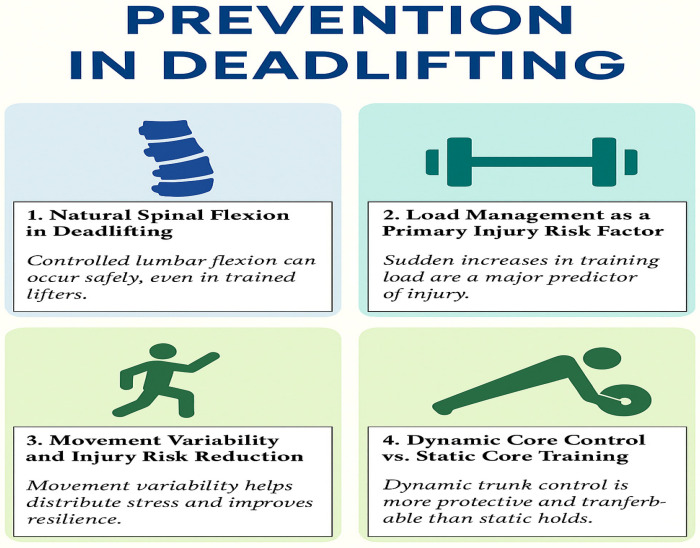
Conceptual framework for low back injury prevention during deadlifting, illustrating hypothesized multidirectional interactions among biomechanical, neuromuscular, load management, and psychological factors. The relationships depicted are theoretical and not intended to represent linear or deterministic causality.

This narrative review re-examines the traditional “neutral spine” paradigm in deadlift coaching by integrating contemporary evidence on spinal flexion, load management, movement variability, dynamic trunk control, and psychological influences. Collectively, the five thematic domains identified highlight the multifactorial nature of low back injury risk and support a shift from rigid postural prescriptions toward a more adaptive and load-informed framework for injury prevention (Figure 1).

### 3.1. Reconsidering the Neutral Spine Paradigm

Maintaining a neutral spinal posture has long been promoted as a universal safeguard against injury during lifting. However, biomechanical and epidemiological findings reviewed earlier, including those reported by Howe and Lehman [28] and Saraceni et al. [9], indicate that moderate lumbar flexion during lifting is not consistently associated with increased injury risk. While this cue may be useful for teaching gross movement patterns, particularly in novice lifters, evidence from biomechanical and epidemiological studies suggests that moderate lumbar flexion during deadlifting is not inherently pathological. Howe and Lehman [31] and Saraceni et al. [9] demonstrated that lumbar flexion is not consistently associated with pain or injury outcomes, challenging the assumption that spinal deviation from neutrality is intrinsically harmful. Furthermore, Schäfer et al. [26] emphasized that the spine experiences complex, multiplanar loading during physical activity and is capable of tolerating variable postures when exposure is progressive and controlled.

The biomechanical evidence reviewed above, including findings from Saraceni et al. [9], Howe and Lehman [31], and Schäfer et al. [29], supports a contextual interpretation of spinal alignment. Rather than prescribing neutrality as an absolute rule, posture should be viewed as one of several modifiable contributors to spinal loading. In certain situations such as during early skill acquisition, in older adults, or near maximal lifting attempts, temporary emphasis on neutral alignment may be beneficial to simplify motor control demands and limit extreme joint excursions [26,44,46]. However, for trained individuals, controlled spinal flexion may represent a functional adaptation that reflects individual anthropometry, mobility, and load distribution strategies rather than technical failure.

Importantly, the present framework does not suggest that spinal posture is irrelevant or that all flexion strategies are equally appropriate under all circumstances. Rather, spinal alignment, load magnitude, fatigue state, and individual tissue capacity likely interact in nonlinear and context-dependent ways [15,16,29]. Excessive or poorly tolerated flexion under high load, particularly in fatigued or deconditioned individuals, may still represent a potential risk factor [8,29]. The present argument is therefore not a rejection of biomechanical principles, but an expansion of them within a broader adaptive and exposure-based model [15,16].

### 3.2. Influence of Lumbar Posture and Spinal Motion

The role of spinal posture during deadlifting remains one of the most debated topics in strength training and injury prevention [37]. Traditional coaching recommendations often emphasize maintaining a rigid neutral spine throughout the lift in order to minimize mechanical stress on lumbar structures [30]. However, emerging biomechanical evidence suggests that spinal posture during deadlifting is not entirely static but rather dynamic throughout the lifting cycle [37].

Experimental studies have shown that increasing external load can lead to measurable increases in thoracic and lumbar spinal flexion during deadlift performance [37]. Similarly, fatigue protocols have been shown to increase trunk range of motion and alter lumbopelvic coordination during repeated deadlift repetitions [34]. These findings suggest that fatigue-related changes in neuromuscular control may influence spinal mechanics during resistance exercise [34].

Importantly, moderate spinal motion during lifting does not necessarily imply an increased injury risk [30]. Instead, the magnitude and control of spinal movement may represent more relevant determinants of spinal tissue stress [35]. Excessive lumbar flexion, particularly when combined with heavy loads or fatigue, may increase shear forces and mechanical stress on spinal structures [6,37]. Therefore, effective neuromuscular control of the lumbopelvic region may be more important than maintaining a perfectly rigid spinal posture throughout the lift [34].

### 3.3. Effect of Deadlift Technique and Exercise Variation

Deadlift technique and exercise variation also appear to influence spinal loading patterns [39]. Comparative biomechanical studies have demonstrated that different deadlift variations produce distinct trunk postures and joint loading patterns [5,40].

For instance, the hex-bar deadlift is typically characterized by a more upright trunk posture compared with the conventional deadlift [5,40]. This more vertical torso position may reduce lumbar flexion and decrease lumbar joint moments during the lift [5]. Similarly, the hex-bar deadlift has been shown to promote a more upright trunk posture and different force distribution compared with the conventional deadlift [40]. These biomechanical differences may shift mechanical demand toward the hip and knee extensors while reducing stress on the lumbar spine [40].

More recent research confirms that technique selection influences how mechanical loads are distributed across the hip and lumbar regions during deadlifting [39]. Therefore, selecting appropriate deadlift variations may represent an effective strategy for managing spinal loading during resistance training [39].

### 3.4. Load Management as the Dominant Injury Driver

Across the literature reviewed above, including the workload–injury models proposed by Gabbett [15] and the IOC consensus summarized by Soligard et al. [13], training load emerges as a more consistent predictor of injury risk than subtle biomechanical variations. Gabbett’s workload–injury model [15], supported by Blanch and Gabbett [41] and Soligard et al. [13], underscores that rapid increases in training volume or intensity elevate injury risk regardless of technical proficiency. In the context of deadlifting, Ramirez et al. [6] quantified spinal compressive forces that approach values that have been proposed as tissue tolerance estimates in experimental and modeling literature, although such thresholds are not universal and likely vary across individuals due to biological variability, training history, and measurement uncertainty. From a practical perspective, this suggests that coaching strategies should prioritize progressive overload, autoregulation, and structured deloading phases rather than exclusive focus on postural cues. Brandl et al. [33] further demonstrated that thoracolumbar fascia deformation differs between individuals with and without back pain during deadlifting, implying that individual tissue responses to load may vary substantially. Consequently, injury prevention strategies should be individualized and load-based rather than universally posture-based.

### 3.5. Movement Variability as an Adaptive Resource

Traditional technical models often conceptualize variability as error, encouraging athletes to reproduce identical movement patterns across repetitions. However, theoretical and empirical work in motor control challenges this view. Stergiou and Decker [17] proposed that optimal movement variability reflects a healthy, adaptable system capable of redistributing mechanical stress. Bartlett et al. [16] further showed that elite performers naturally exploit variability to adapt to task constraints and environmental demands.

In deadlifting, subtle fluctuations in trunk inclination, hip strategy, or bar path may reduce repetitive loading of identical tissue regions, thereby mitigating overuse mechanisms. Bengtsson et al. [30] reported sex-based differences in thoracolumbar and lumbopelvic alignment, reinforcing that technique variability may reflect individual structural and functional characteristics rather than poor execution. These findings suggest that allowing controlled variability—through changes in stance width, tempo, or grip may enhance long-term tissue resilience without compromising performance.

### 3.6. Dynamic Core Control Versus Static Stability

Conventional core training paradigms emphasize static stabilization exercises, such as planks and bracing drills, as prerequisites for safe lifting. While these exercises increase trunk muscle activation, their transfer to dynamic lifting tasks remains uncertain. Behm et al. [23] and Granacher et al. [24] demonstrated that dynamic and perturbation-based exercises elicit greater neuromuscular engagement under movement conditions than static holds. Huxel Bliven and Anderson [21] further conceptualized core stability as the neuromuscular ability to maintain spinal control under load and perturbation rather than as rigid immobility.

These insights support a shift toward task-specific trunk training that mimics the mechanical and coordinative demands of deadlifting. Anti-rotation drills, loaded carries, and reactive trunk exercises may therefore provide more relevant stimuli for enhancing spinal robustness than isolated isometric contractions. Such an approach aligns with the broader conceptual model proposed in this review, which emphasizes adaptability and dynamic control over postural rigidity.

### 3.7. Psychological and Coaching Influences

Psychological beliefs about spinal fragility exert a substantial influence on movement behavior. O’Sullivan et al. [25] and Liew et al. [26] showed that fear-avoidance beliefs are associated with altered motor strategies, increased muscular co-contraction, and reduced movement efficiency. Coaching cues that frame spinal flexion as dangerous may inadvertently promote hypervigilance and excessive bracing, potentially increasing spinal compression and fatigue.

Conversely, Scott et al. [27] demonstrated that resiliency-focused spine education improves both deadlift performance and beliefs about back health. These findings suggest that coaching language plays a mechanistic role in shaping motor output and injury risk. Emphasizing intent-based cues (e.g., “push the ground away,” “move smoothly,” “breathe and lift”) may facilitate efficient force transfer while reducing fear-driven stiffness.

### 3.8. Toward an Integrative Framework

**Figure 2:**
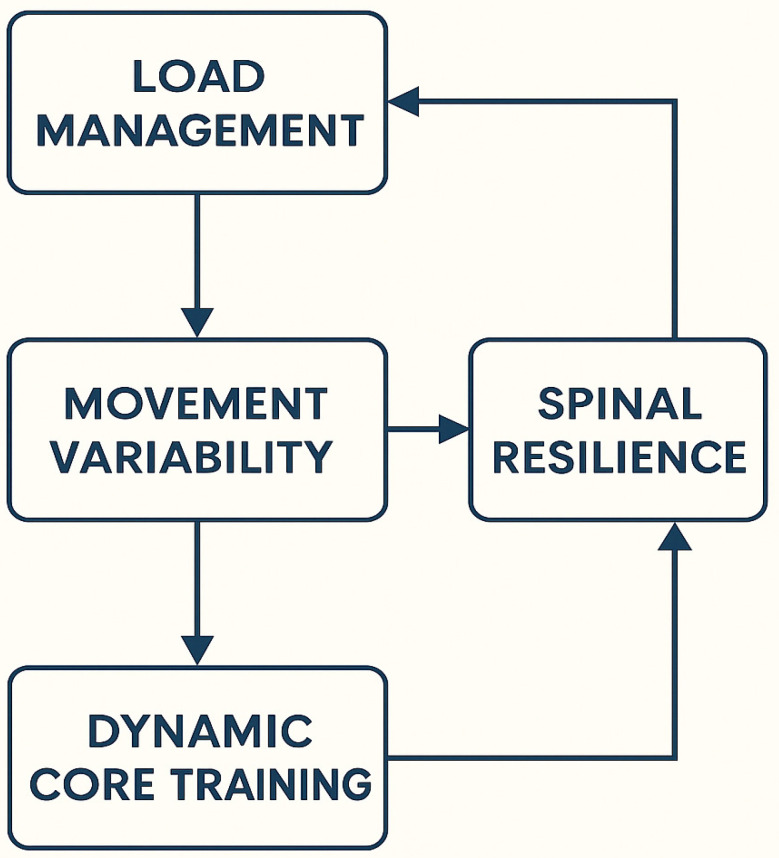
Conceptual hypothesis model illustrating multidimensional and context-dependent interactions contributing to injury risk and resilience in deadlifting.

The present findings support a multifactorial model of injury risk in deadlifting, in which mechanical loading, neuromuscular adaptability, and cognitive–behavioral factors interact dynamically [13,14]. This biopsychosocial framework reframes spinal health as an emergent property of load tolerance, movement variability, and psychological readiness rather than as strict adherence to neutral alignment (Figure 2). Within this framework, psychological constructs such as fear-avoidance, self-efficacy, and coaching language are not peripheral variables but modulators of motor strategy and load distribution [41]. Beliefs about spinal fragility may increase co-contraction and stiffness, thereby altering mechanical exposure patterns. Consequently, psychological and biomechanical processes are reciprocally linked within a multidimensional injury model.

Practically, this model implies that injury prevention should prioritize:

1. progressive and individualized load management,
2. acceptance of controlled movement variability,
3. dynamic and task-specific trunk training, and
4. coaching strategies that promote confidence and autonomy.

Such an approach recognizes the spine as a load-bearing, adaptable structure rather than as an inherently vulnerable segment requiring rigid protection.

### 3.9. Practical Implications for Injury Prevention

Taken together, the evidence summarized in Table 1, Table 2 and Table 3 suggests that lumbar injury risk during deadlifting is influenced by multiple interacting factors including load magnitude, spinal posture, fatigue, and lifting technique [34,37]. Rather than focusing exclusively on maintaining a perfectly neutral spine, injury prevention strategies should consider broader aspects of lifting mechanics and training design [30].

First, progressive load management is essential, as spinal compression and shear forces increase substantially with heavier external loads [6]. Second, technical proficiency and neuromuscular control should be emphasized to ensure coordinated movement between the hips and lumbar spine during lifting [34]. Third, monitoring fatigue and training volume may help reduce compensatory movement patterns that could increase spinal stress [34].

Finally, selecting appropriate deadlift variations based on individual anthropometrics, mobility, and training experience may help distribute mechanical load more effectively across the kinetic chain [39,40]. In this context, the concept of a “neutral spine” may be better interpreted as a controlled and stable spinal position rather than a completely rigid posture during dynamic lifting tasks [30].

### 3.10. Limitations and Future Directions

As a narrative review, this work does not provide a systematic or quantitative synthesis of the literature and is therefore subject to selection and interpretive bias. The heterogeneity of study designs and outcome measures also limits direct causal inference. Additionally, much of the current evidence is derived from laboratory-based biomechanical studies or cross-sectional designs, which may not fully capture long-term injury mechanisms in real-world training contexts.

Future research should aim to empirically test the proposed framework through longitudinal and experimental studies examining (i) individual tolerance to spinal flexion under load, (ii) the long-term effects of variability-based lifting strategies, and (iii) the comparative effectiveness of static versus dynamic trunk training for injury prevention. Investigations incorporating psychological constructs such as fear-avoidance and self-efficacy may further clarify the role of cognitive factors in lifting-related injury risk.

## 4. Conclusions

This narrative review re-examines the traditional belief that any deviation from a neutral spine during deadlifting inherently increases injury risk. Emerging evidence suggests that moderate lumbar flexion, when progressively exposed and appropriately tolerated, may represent a functional adaptation in trained individuals, although long-term prospective evidence remains limited. More importantly, injury risk appears to be more closely associated with poor load management, insufficient movement variability, and limited dynamic trunk control. Shifting the focus from rigid technique enforcement to a holistic view of training variables may help coaches and clinicians better protect athletes while enhancing performance. This multifactorial, evidence-informed framework encourages a nuanced approach to deadlift programming that mirrors real-world demands. By embracing progressive loading, movement adaptability, dynamic core control, and resiliency-focused coaching, practitioners can use deadlifting not only as a performance tool but also as a means of developing robust, confident spinal function. Future research should aim to empirically validate this model across diverse populations and lifting contexts.

## Figures and Tables

**Table 1:** Experimental Studies Investigating Deadlift Biomechanics and Lumbar Spine Loading.

Study	Participants (Sex, Number)	Training/ Level	Deadlift Type	Load/Protocol	Measurement Method	Biomechanical Variables	Key Quantitative Results
[30]	24 participants (14 men and 10 women)	Strength-trained	Barbell deadlift	Controlled repetitions	Inertial measurement units	Thoracolumbar and lumbopelvic alignment	Deadlift execution involved dynamic changes in lumbar alignment
[34]	23 participants (18 male and 5 female)	Recreational powerlifters	Hex-bar deadlift	Repetitions-to-failure (68 kg)	3D motion capture	Lumbopelvic coordination, trunk ROM	Fatigue increased trunk and pelvic ROM during repetitions
[35]	1 participant (male)	Healthy trained adults	Conventional deadlift	68 kg lift	Musculoskeletal modeling	Trunk muscle forces, spinal loads	Personalized modeling altered predicted L5-S1 loads and muscle forces
[36]	10 participants (female)	Strength-trained	Conventional deadlift	3 RM of deadlift	Motion capture + EMG	Thoracic/lumbar flexion, joint moments	Increased fatigue led to greater thoracic/lumbar flexion and higher erector spinae activation
[37]	13 participants (male)	University track and field athletes	Conventional deadlift	Progressive load conditions	Linked-segment motion analysis	Segmental spinal flexion	Load increases produced greater lower-thoracic and upper-lumbar flexion
[38]	20 participants (male)	Post-secondary students	Lifting task (deadlift-like)	Various loads and heights	Motion capture	Mechanical energy distribution	Restricting lumbar flexion shifted mechanical work toward hips
[39]	30 participants (male)	Healthy and physically active	Conventional vs. sumo deadlift	Controlled lifting	Kinematics and EMG	Joint moments, muscle activation	Technique differences alter hip and lumbar loading patterns

**Table 2:** Experimental Evidence Linking Lumbar Posture and Spinal Loading During Deadlift-Type Lifting.

Study	Participants (Sex, Number)	Training/ Level	Lifting Task	Lumbar Posture Condition	Measurement Method	Biomechanical Variables	Key Quantitative Findings	Injury-Risk Interpretation
[30]	24 participants (14 men and 10 women)	Strength-trained	Barbell deadlift	Natural vs. lifting posture	Inertial sensors	Thoracolumbar and lumbopelvic alignment	Significant changes in lumbar angles during lift phases	Lumbar posture changes are common during heavy lifts
[34]	23 participants (18 male and 5 female)	Recreational powerlifters	Hex-bar deadlift	Fatigue-induced posture changes	3D motion capture	Lumbopelvic coordination	Repetition-to-failure increased trunk and pelvis ROM	Fatigue alters spinal control and may increase injury risk
[36]	10 participants (female)	Strength-trained	Conventional deadlift	70–100% 3 RM	Motion capture + EMG	Spinal kinematics and muscle activation	Higher loads increased spinal flexion and erector spinae activation	Heavy loads increase spinal mechanical stress
[37]	13 participants (male)	University track and field athletes	Deadlift	Increasing load conditions	Segmental spinal modeling	Thoracic and lumbar flexion	Greater load increased lower-spine flexion ROM	Load-induced flexion may increase tissue stress
[38]	20 participants (male)	Post-secondary students	Lifting task	Restricted vs. unrestricted lumbar motion	Motion capture	Mechanical energy expenditure	Restricting lumbar flexion increased hip contribution	Neutral spine redistributes loads away from lumbar spine

**Table 3:** Comparison of Lumbar Loading Across Deadlift Variations.

Study	Participants (Sex, Number)	Training Level	Deadlift Variation Compared	Load/Protocol	Measurement Method	Key Biomechanical Variables	Main Findings	Injury-Risk Interpretation
[5]	19 participants (male)	Competitive powerlifters	Conventional vs. Hex-bar	~70% 1 RM	Motion capture + force plates	Joint moments, trunk inclination	Hex-bar deadlift showed more upright trunk posture and lower lumbar moments	May reduce lumbar loading
[30]	Men and women	Strength-trained	Barbell deadlift	Submaximal loads	Inertial sensors	Thoracolumbar alignment	Lumbar posture changed dynamically during lift	Lumbar posture changes dynamically during deadlifting, indicating variability in spinal alignment across lift phases
[34]	24 participants (14 men and 10 women)	Recreational powerlifters	Hex-bar deadlift fatigue protocol	Repetitions-to-failure	Motion capture	Lumbopelvic coordination	Fatigue increased trunk ROM	Fatigue increased trunk range of motion and lumbopelvic variability, which may influence spinal loading patterns
[39]	30 participants (male)	Healthy and physically active	Conventional vs. Sumo	Controlled lifting trials	Motion capture + EMG	Hip and lumbar joint moments	Sumo deadlift showed a more upright trunk posture and altered hip and lumbar joint moments compared with conventional deadlift	Technique selection influences spinal stress
[40]	20 participants (male)	Resistance-trained	Conventional vs. Hex-bar	70% 1 RM	Force plates + motion capture	Ground reaction force, trunk angle, muscle activation	Hex-bar deadlift produced more upright trunk posture and greater power output	Reduced lumbar stress

## Data Availability

Not applicable.

## Abbreviations

ACWR	acute:chronic workload ratio
